# Supplementary material for: An original potentiating mechanism revealed by the cryo-EM structures of the human α7 nicotinic receptor in complex with nanobodies
Source: Nat Commun. 2023 Sep 25;14:5964. doi: 10.1038/s41467-023-41734-4 (PMC10520083; doi:10.1038/s41467-023-41734-4)
Supplement: Supplementary file 1 — Supplementary Information [file 41467_2023_41734_MOESM1_ESM.pdf]

Supplementary Figures and Tables

An original potentiating mechanism revealed by the cryo-EM structures of the human α7 nicotinic receptor in complex with nanobodies

PREVOST Marie S.[1#*], BARILONE Nathalie[1#], DEJEAN DE LA BATIE Gabrielle[1], PONS Stéphanie[2], AYME Gabriel[3], ENGLAND Patrick[4], GIELEN Marc[1,5], BONTEMS François[6,7], PEHAU-ARNAUDET Gérard[8], MASKOS Uwe[2], LAFAYE Pierre[3], CORRINGER Pierre-Jean[1*]

1 Institut Pasteur, Université Paris Cité, CNRS UMR 3571, Channel-Receptors Unit, Paris, France
2 Institut Pasteur, Université Paris Cité, CNRS UMR 3571, Integrative neurobiology of cholinergic systems Unit, Paris, France
3 Institut Pasteur, Université Paris Cité, CNRS UMR 3528, Antibody Engineering platform, Paris, France
4 Institut Pasteur, Université Paris Cité, CNRS UMR 3528, Molecular Biophysics plarform, Paris, France
5 Sorbonne Université, Paris, France
6 Institut Pasteur, Université Paris Cité, CNRS UMR 3569, Structural Virology unit, Paris, France
7 Institut de chimie des substances naturelles, Centre national de la recherche scientifique, Université Paris Saclay, Gif-sur-Yvette, France
8 Institut Pasteur, Université Paris Cité, Ultrastructural Bioimaging Core facility, Paris, France

# equal contribution
* Corresponding authors
Email:
marie.prevost@pasteur.fr
pierre-jean.corringer@pasteur.fr

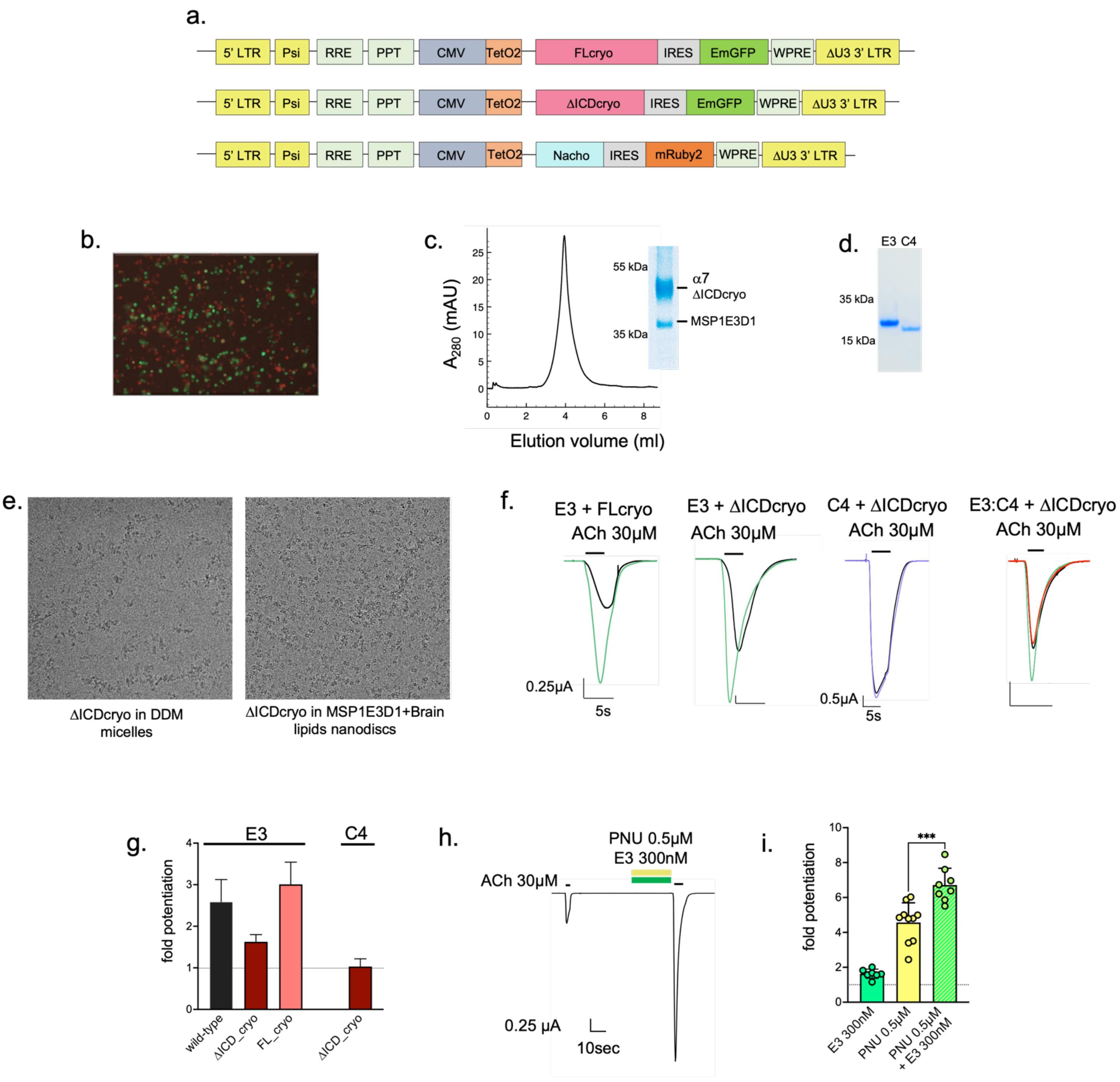

Supplementary figure 1: Production of the α7 nAChR and functional characterization of E3 WT and mutants

a. Lentiviral transfer regions used in the study. LTR: Long Terminal Repeat sequence, ΔU3: Deletion in 3'LTR rendering the virus "self-inactivating" (SIN) after integration, Psi : Packaging signal sequence ; RRE: Rev Response Element cPPT: Central Polypurine Tract ; CMV: Cytomegalovirus Promoter ; TetO2 : Tetracycline-controlled transcriptional activation sequence ; IRES : Internal ribosome entry site of the encephalomyocarditis virus; WPRE: Woodchuck Hepatitis Virus Post-transcriptional Regulatory Element
b. After transduction and induction, most cells exhibit orange and green fluorescence indicating efficient cell infection.
c. Size-exclusion profile of α7ΔICDcryo reconstituted in nanodiscs and SDS PAGE of the concentrated sample before plunge freezing.
d. SDS-PAGE of E3 and C4 after purification.

e. Representative micrographs of α7ΔICDcryo in detergent micelles or reconstituted in nanodiscs, showing the presence of aggregates in the former.
f. Representative TEVC recordings showing the potentiation of 30µM ACh-elicited currents upon a 30s pre-application of 1µM of E3 (green) or C4 (purple) or by 0.25µM E3 and 0.5µM C4 (red) with the indicated α7 constructs.
g. Fold potentiation of 30µM ACh currents determined with a 30sec pre-application of 1µM nanobody for the indicated constructs. Data are mean ±sd for n≥4.
h. Representative trace of a co-pre-application of E3 and PNU-120596 (PNU).
i. Fold potentiation of 30µM ACh currents determined with a 30sec pre-application of 300nM E3, 0.5µM PNU or 300nM E3 and 0.5µM PNU. Data are mean ±sd for n≥8.

```
                  Signal peptide          1  Nter helix          pre-β1
WT         MRCSPGGVWLALAASLLHVSLQGEFQRKLYKELVKNYNPLERPVANDSQPLTVYF
FL_cryo    MRCSPGGVWLALAASLLHVSLQGEFQRKLYKELVKNYNPLERPVANDSQPLTVYF
ΔICD_cryo  MRCSPGGVWLALAASLLHVSLQGEFQRKLYKELVKNYNPLERPVANDSQPLTVYF

                                           MIR
           33
WT         SLSLLQIMDVDEKNQVLTTNIWLQMSWTDHYLQWNVSEYPGVKTVRFPDGQIWKP
FL_cryo    SLSLLQIMDVDEKNQVLTTNIWLQMSWTDHYLQWNVSEYPGVKTVRFPDGQIWKP
ΔICD_cryo  SLSLLQIMDVDEKNQVLTTNIWLQMSWTDHYLQWNVSEYPGVKTVRFPDGQIWKP

           88
WT         DILLYNSADERFDATFHTNVLVNSSGHCQYLPPGIFKSSCYIDVRWFPFDVQHCK
FL_cryo    DILLYNSADERFDATFHTNVLVNSSGHCQYLPPGIFKSSCYIDVRWFPFDVQHCK
ΔICD_cryo  DILLYNSADERFDATFHTNVLVNSSGHCQYLPPGIFKSSCYIDVRWFPFDVQHCK

           143
WT         LKFGSWSYGGWSLDLQMQEADISGYIPNGEWDLVGIPGKRSERFYECCKEPYPDV
FL_cryo    LKFGSWSYGGWSLDLQMQEADISGYIPNGEWDLVGIPGKRSERFYECCKEPYPDV
ΔICD_cryo  LKFGSWSYGGWSLDLQMQEADISGYIPNGEWDLVGIPGKRSERFYECCKEPYPDV

           198
WT         TFTVTMRRRTLYYGLNLLIPCVLISALALLVFLLPADSGEKISLGITVLLSLTVF
FL_cryo    TFTVTMRRRTLYYGLNLLIPCVLISALALLVFLLPADSGEKISLGITVLLSLTVF
ΔICD_cryo  TFTVTMRRRTLYYGLNLLIPCVLISALALLVFLLPADSGEKISLGITVLLSLTVF

           253
WT         MLLVAEIMPATSDSVPLIAQYFASTMIIVGLSVVVTVIVLQYHHHDPDGGKMPKW
FL_cryo    MLLVAEIMPATSDSVPLIAQYFASTMIIVGLSVVVTVIVLQYHHHDPDGGKMPKW
ΔICD_cryo  MLLVAEIMPATSDSVPLIAQYFASTMIIVGLSVVVTVIVLQYHHHDPDGGKMPKW

           308
WT         TRVILLNWCAWFLRMKRPGEDKVRPACQHKQRRCSLASVEMSAVAPPPASNGNLL
FL_cryo    TRVILLNWCAWFLRMKRPGEDKVRPACQHKQRRCSLASVEMSAVAPPPASNGNLL
ΔICD_cryo  TRVILLNWCAWFLRMKR--------------------------------------

           363
WT         YIGFRGLDGVHCVPTPDSGVVCGRMACSPTHDEHLLHGGQPPEGDPDLAKILEEV
FL_cryo    YIGFRGLDGVHCVPTPDSGVVCGRMACSPTHDEHLLHGGQPPEGDPDLAKILEEV
ΔICD_cryo  -------DGV---------------------------------------------

           418
WT         RYIANRFRCQDESEAVCSEWKFAACVVDRLCLMAFSVFTIICTIGILMSAPNFVE
FL_cryo    RYIANRFRCQDESEAVCSEWKFAACVVDRLCLMAFSVFTIICTIGILMSAPNFVE
ΔICD_cryo  --------------AVCSEWKFAACVVDRLCLMAFSVFTIICTIGILMSAPNFVE

           473
WT         AVSKDFA-------------
FL_cryo    AVSKDFASAGLTETSQVAPA
ΔICD_cryo  AVSKDFASAGLTETSQVAPA
                   linker Rho1D4
```

X Mutated residue in this study

X Residue not visible in the structures

Supplementary figure 2: Sequence alignment of human α7-nAChRs and the constructs used in this study.

Signal peptide regions are denoted in green. A box locates the residues mutated in this study. Grey residues form the transmembrane and intracellular regions not resolved in the structures.

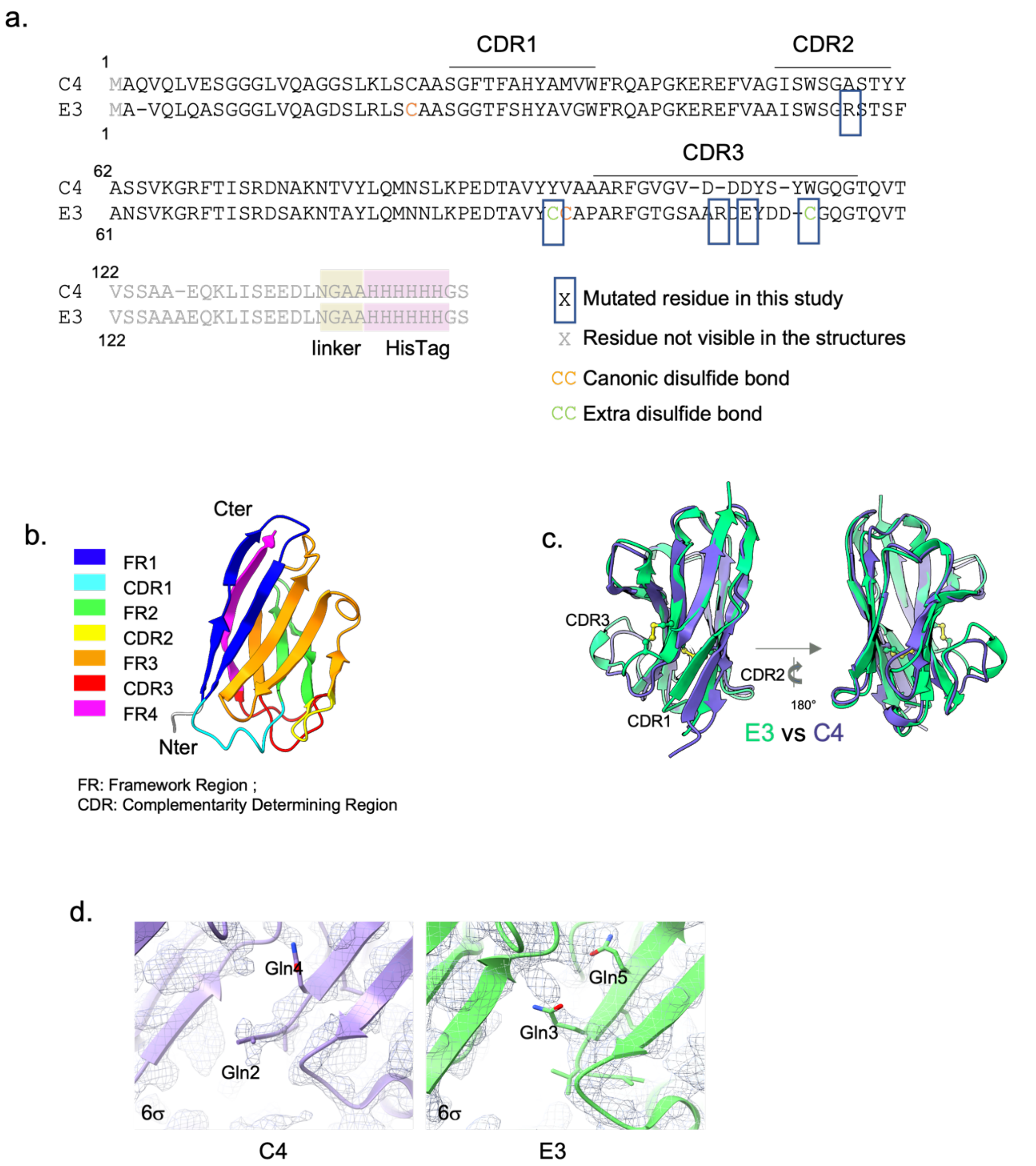

Supplementary figure 3: Sequence and structure of nanobodies E3 and C4

a. Sequence alignment of E3 and C4. A box locates the residues mutated in this study. The cysteines involved in the canonical and extra disulfide bonds are denoted in orange and green respectively. Grey residues are not resolved in the structures. Note the missing Gln at the N-terminus of E3 that shifts the numbering of the whole nanobody compared to C4.
b. Topology of the nanobodies exemplified with the structure of C4. Elements are rainbow-colored from the Nter (blue) to Cter (pink).
c. Tertiary structure comparison of C4 (purple) and E3 (green). The two disulfide bonds of E3 are shown in sticks representation.
d. The N-terminus of C4 extends towards the solvent while the N-terminus of E3 folds back inside the β-sandwich. Close-view of the Nter of the two nanobodies as seen in the C4-Apo and E3-Apo datasets and their corresponding electron densities contoured at 6σ. The two aligned glutamine residues are shown in sticks.

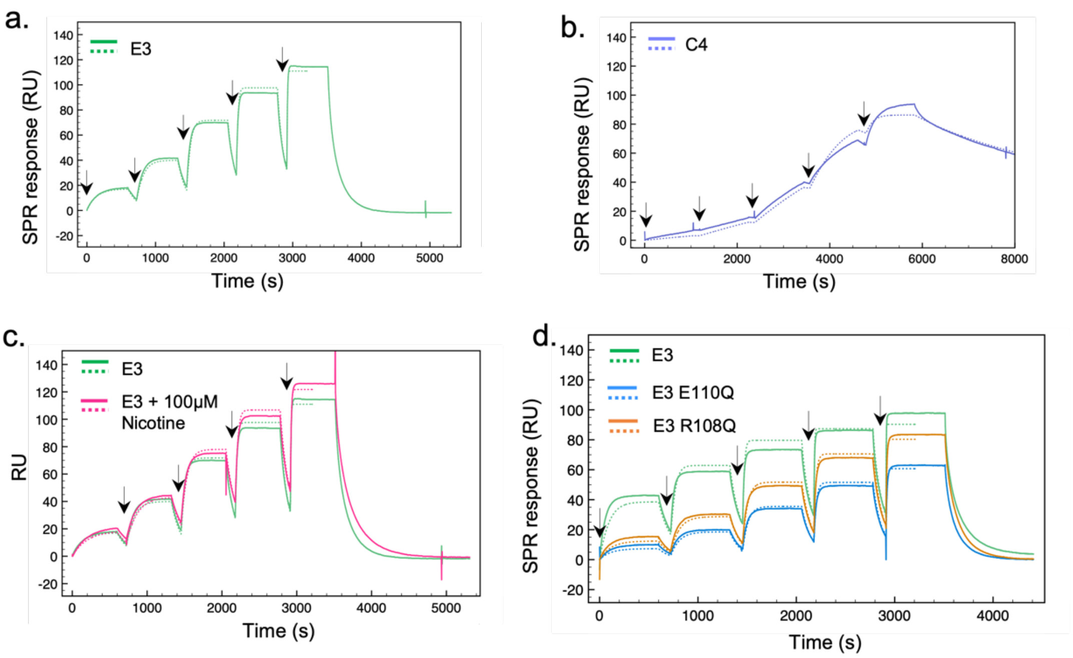

e.

	α7 prep 1		α7 prep 2			
	E3 WT	E3 WT + 100μM Nicotine	E3 WT	E3 E110Q	E3 R56A	C4
k_{on} (10^6 $M^{-1}.s^{-1}$)	1.6 ±0.6	1.2 ±0.5	11 ±0.02	18.2 ±0.06	7.0 ±0.15	1.2 ±0.05
k_{off} (10^{-2} s^{-1})	1.3 ±0.5	0.85 ±0.3	1.9 ±0.01	1.1 ±0.01	1.0 ±0.02	0.024 ±0.002
Kd (nM)	8.0 ±3	7.4 ±0.3	1.0 ±0.01	0.62 ±0.02	1.5 ±0.01	0.20 ±0.26
Rmax	137 ±50	151 ±22	90 ±2	65 ±1	86 ±1	78 ±14
Chi^2	5.9 ±0.5	12 ±1.9	23.2 ±0.8	5.3 ±0.01	5.4 ±0.1	5.1 ±2.7

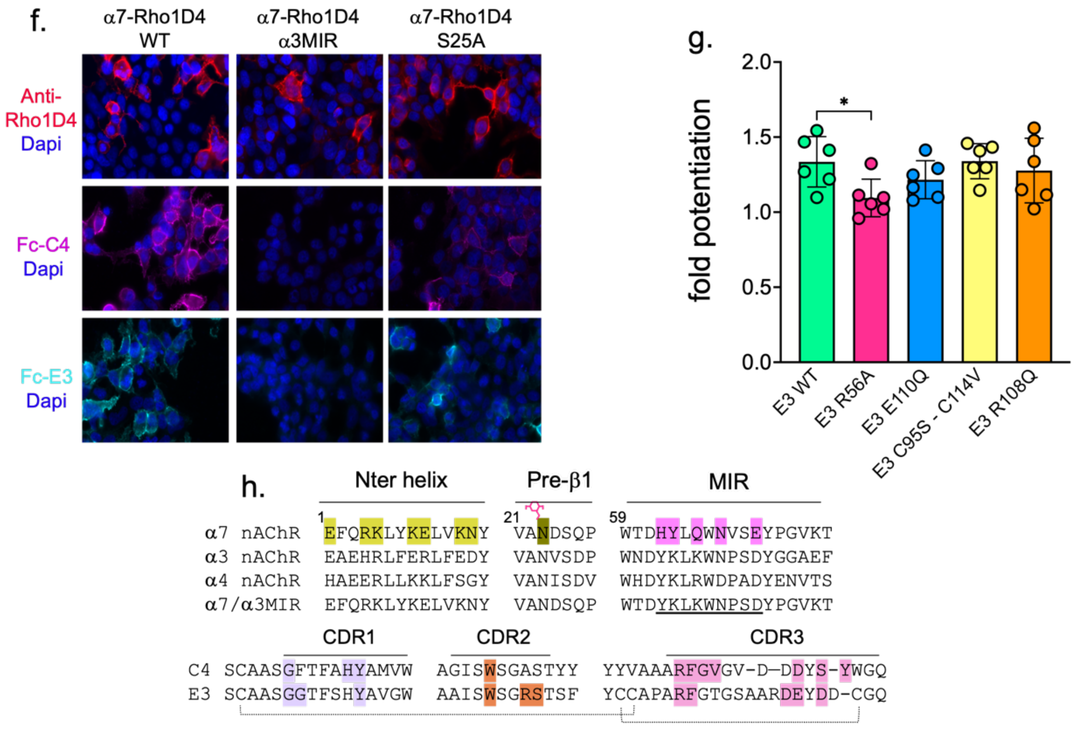

Supplementary figure 4: characterization of the α7 nAChR, C4, E3 and their mutants by SPR, immunofluorescence and electrophysiology

a. Real-time SPR monitoring of injections of increasing concentrations of E3 (black arrows) on purified α7ΔICDcryo reconstituted in nanodiscs. The dashed line shows the fitting curve used to determine the kinetic constants.

b. Same as in a. with C4.
c. Same as in a. with E3 (green) and E3 with 100µM nicotine in the running buffer (pink).
d. Same as in a with E3 (green), E3 E110Q (blue) and E3 R108Q (orange).
e. Kinetic and affinity constants determined by single cycle kinetics surface plasma resonance. E3 WT was first assayed with or without 100µM nicotine on a single α7ΔICDcryo nanodiscs preparation. Another preparation was used to perform the assay with E3 WT and its mutants and with C4. Data are mean ±sd of 3 independent channels on the sensorchip. Chi^2 values were determined during analysis and reflect the goodness of the fit.
f. Immunofluorescence of C4 and E3 fused to Fc on HEK cells expressing α7 WT, S25A or the α7/α3MIR chimera. Top: α7 receptors are labelled using the Cter Rho1D4 tag (red), nuclei using Dapi (blue). Middle: Fc-C4 labelling is shown in purple, nuclei are labelled using Dapi (blue). Bottom: Fc-E3 labelling is shown in green, nuclei are labelled using Dapi (blue).
g. Fold potentiation calculated by TEVC on n≥4 cells by E3-WT and its mutants on α7-WT. Values were submitted to a paired t-test that is denoted with * $p \leq 0.05$ when a significant difference was found.
h. Sequence alignments of the loops involved in binding. Top: alignment of the Nter helix, pre-β1 and MIR of α7, α3, α4 and the chimeric α7/α3MIR construct (with the chimeric sequence underlined). Key residues involved in binding are highlighted and the glycan on Asn23 depicted as a pink sugar. Sequence numbering is the one of α7. Bottom: alignments of the three CDR regions of C4 and E3. Key residues involved in binding are highlighted. Disulfide bonds of E3 are depicted by links between the two cysteines. CDR3s alignment is based on their local structure.

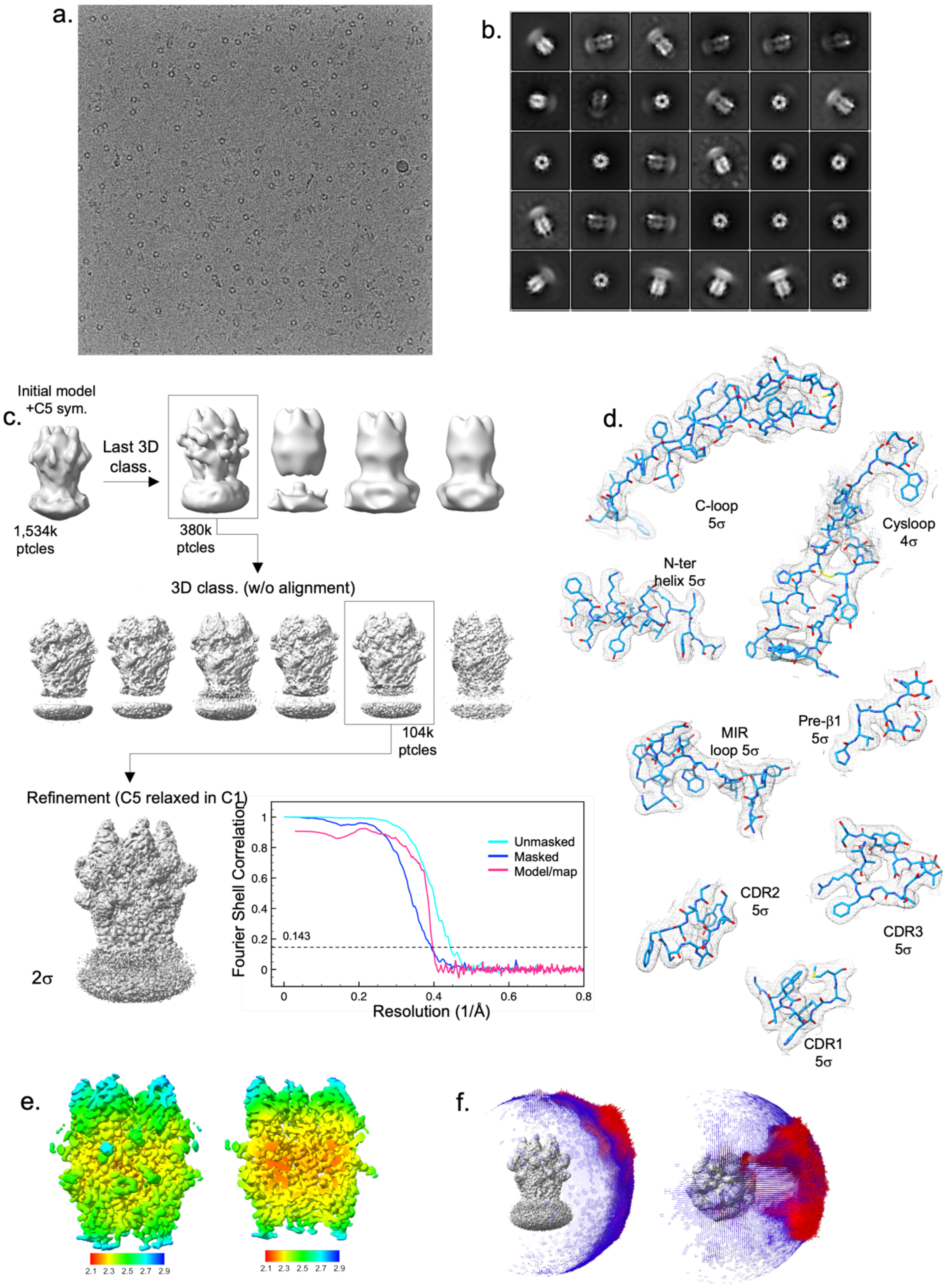

Supplementary figure 5: Electron microscopy and 3D reconstruction of the C4-Apo data set

a. Representative micrograph of the C4-Apo dataset.
b. Selected 2D class averages
c. Flowchart of the 3D volume processing. A C5-symmetry was applied on the initial model for 3D classification with particles alignment. The best class particles were submitted to another 3D classification round without alignment and high-resolution reaching particles further refined with a

C5 symmetry relaxed in C1 (Relion 3D refine). Resulting volume is shown at 2σ contouring with the FSC curves on its right.

d. Representative densities and model building for the loop C, cys-loop, Nter helix, pre-β1, MIR of α7 and the three CDRs of C4 with the indicated contouring level

e. Local resolution map calculated with Relion. The volume is shown at high contouring from the side and sliced in the vestibule.

f. Angular distribution of the particles seen on the side and top views of the unsharpened map

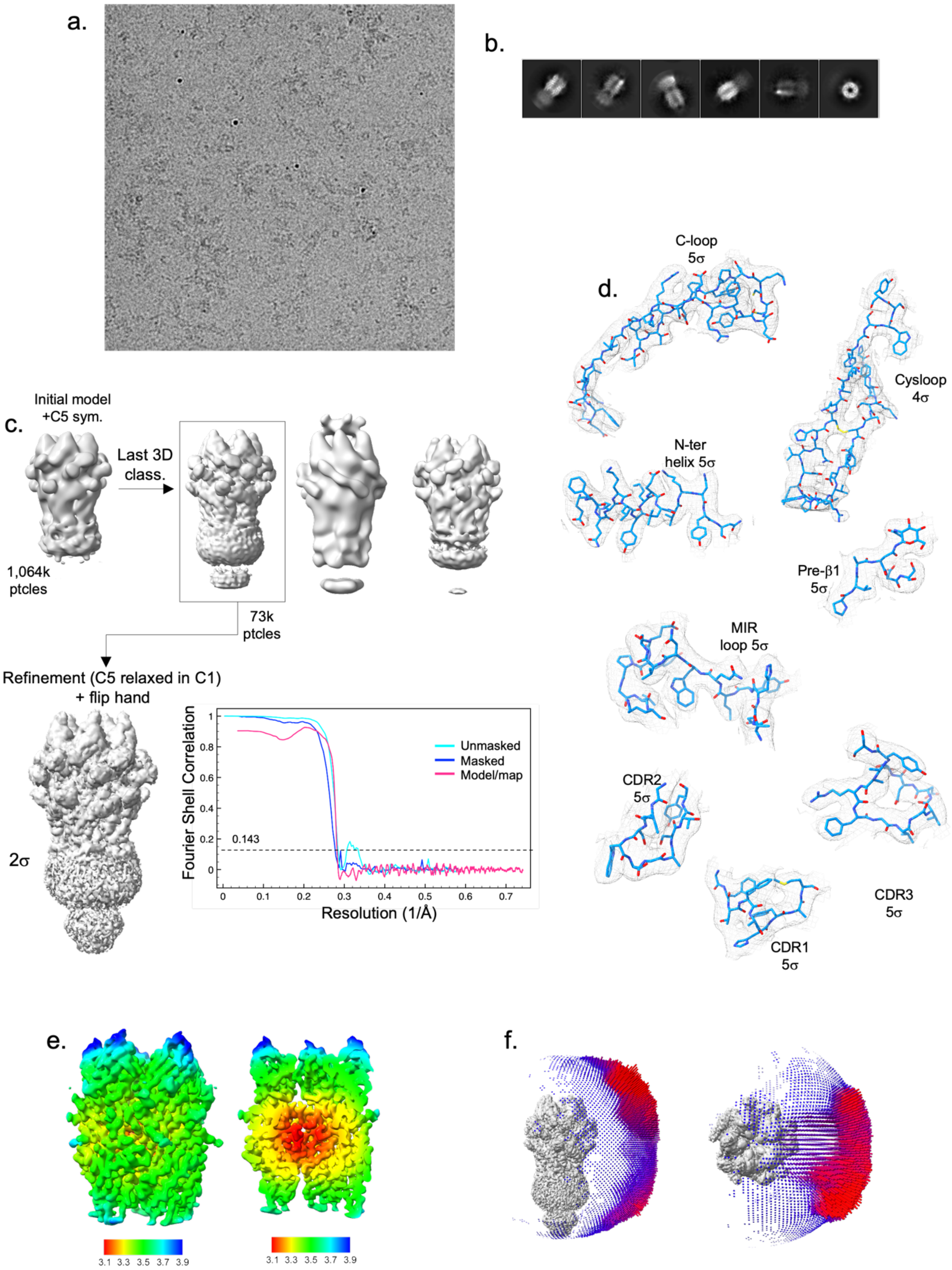

Supplementary figure 6: Electron microscopy and 3D reconstruction of the C4-Nic data set

a. Representative micrograph of the C4-Nic dataset.
b. Selected 2D class averages
c. Flowchart of the 3D volume processing. A C5-symmetry was applied on the initial model for 3D classification with particles alignment. The best class particles were further refined with a C5 symmetry relaxed in C1 (Relion 3D refine). Resulting volume is shown at 2σ contouring with the FSC curves on its right.

d. Representative densities and model building for the loop C, cys-loop, Nter helix, pre-β1, MIR loop of α7 and the three CDR of C4 with the indicated contouring level
e. Local resolution map calculated with Relion. The volume is shown at high contouring from the side and sliced in the vestibule.
f. Angular distribution of the particles seen on the side and top views of the unsharpened map

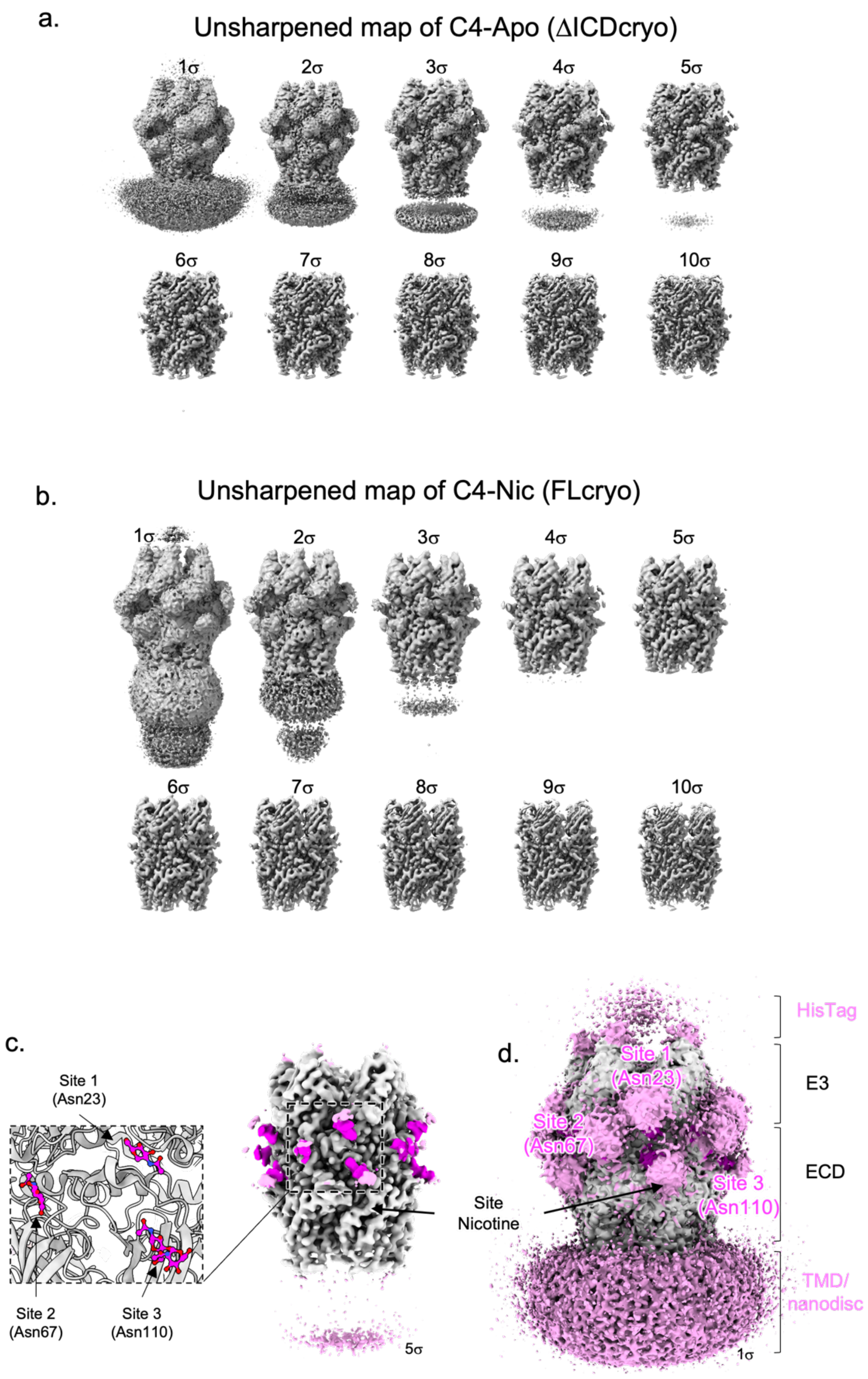

Supplementary figure 7: **Unsharpened cryo-EM maps of C4-Apo and C4-Nic and glycosylation sites**

a. Various contouring levels applied to the C4-Apo density showing the flexibility of the transmembrane/nanodisc region that completely disappears at 6σ.
b. Various contouring levels applied to the C4-Nic density showing the flexibility of the transmembrane/nanodisc and intracellular regions that completely disappear at 4σ.
c. Glycosylation sites resolved in the E3-Apo density (at 5σ) with a close-up view of the built glycan. Regions where protein is built is in grey, built glycans in hot pink and unbuilt regions in light pink.
d. Same as in c. but with a contouring level of 1σ.

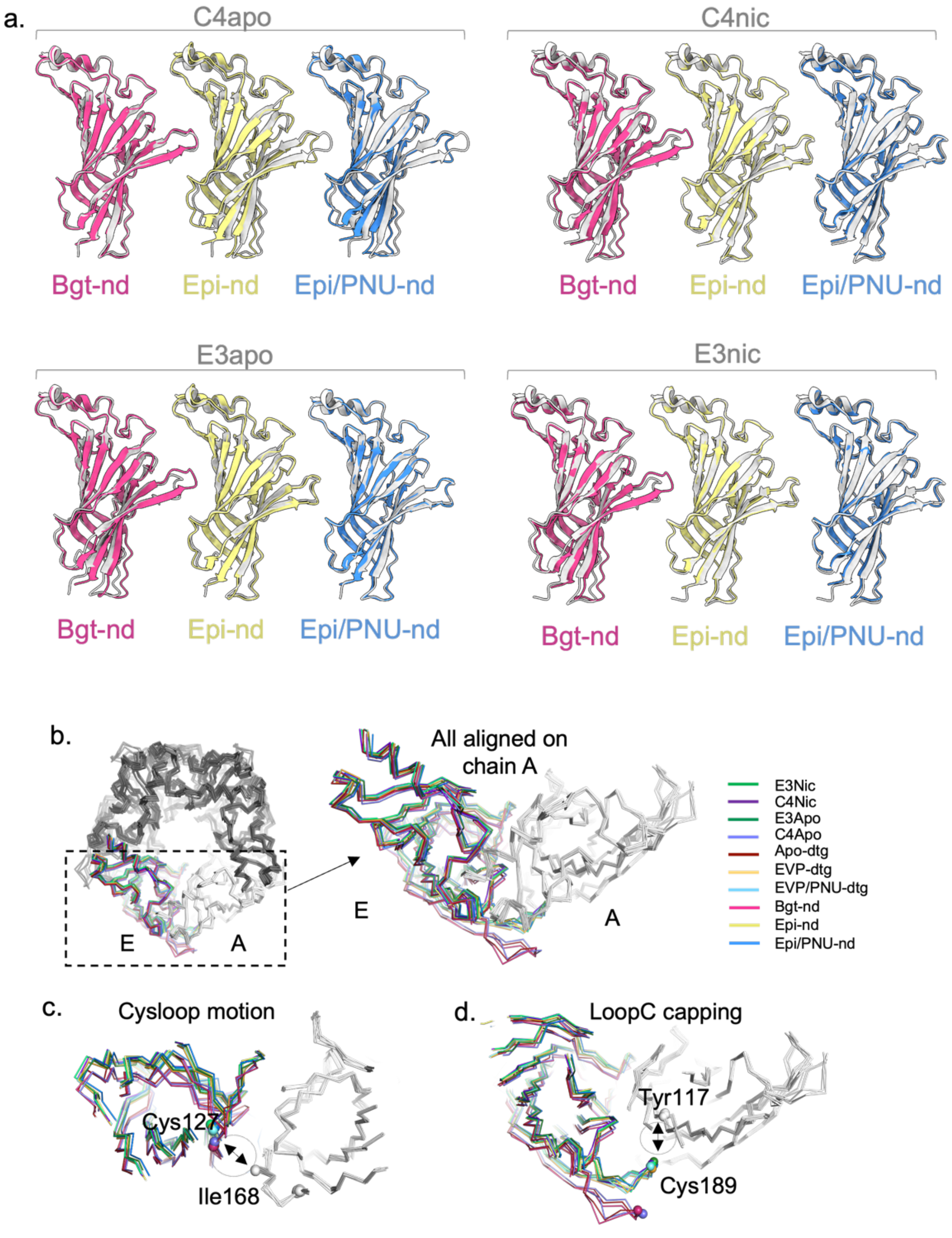

e.

	E3Apo	E3Nic	C4Nic	C4Apo	$C4_{partial}Apo$
α7:α7	1275 ±14	1245 ±2	1252 ±9	1212 ±5	1128 ±37
E3/C4:α7	511 ±2	486 ±1	472 ±2	506 ±2	504 ±45
E3/C4:$α7_{n+1}$	300 ±5	278 ±1	261 ±5	293 ±2	278 ±7
E3/C4:E3/$C4_{n+1}$	213 ±7	216 ±1	213 ±4	198 ±1	259*

Supplementary figure 8: Analysis of the conformation of the α7 ECD in E3 and C4 co-structures

a. Comparisons of the ECD conformations with the previously solved nanodiscs structures. Whole ECD pentamers where used for alignment and a single subunit is shown. In all four panels, the Bgt-nd structure is in pink, the Epi-nd in yellow and the Epi/PNU-nd in blue, while C4/E3-bound structures are in white.
b. α7 pentamers from known structures are aligned at the level of their chain A and represented as ribbons in top view. On the right, a dimer A-E of the aligned structures is shown. Chains A are seen in grey and chains E according to the color-coding on the right.

c. Same as in b. but clipped to reveal the cys-loop and its motion towards the chain A. The “cys-loop motion” distance measurement between the Cα of Cys127 and Ile 168 is figured with an arrow.
d. Same as in b. but clipped to reveal the Loop C and its motion towards the chain A. The “Loop C capping” distance measurement between the Cα of Cys189 and Tyr117 is figured with an arrow.
e. Interaction surface areas calculated using PISA (CCP4 suite) in $Å^2$. Values are mean ±sd calculated from each chain. *a single C4:C4 interface is present for this structure

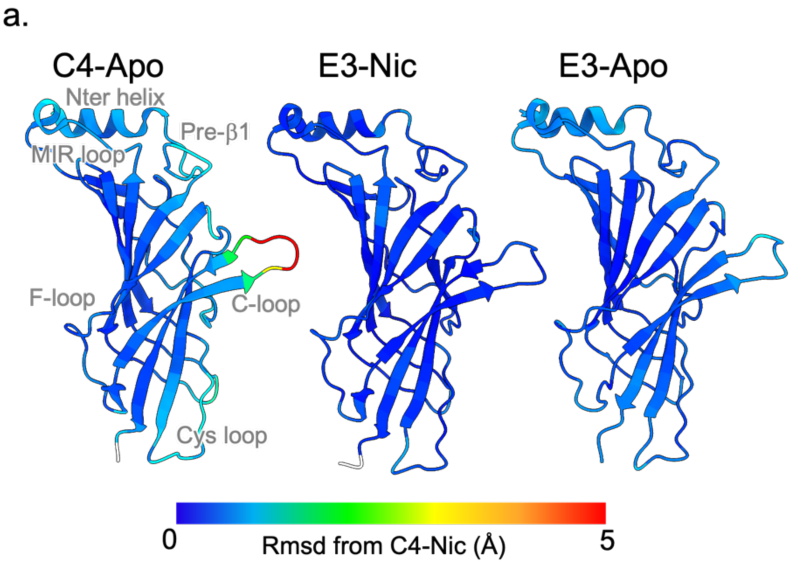

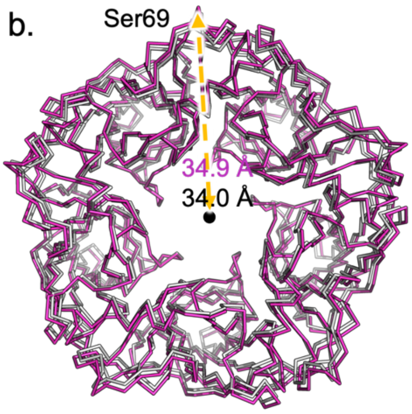

structure	distance (Å)
E3Apo	34.0
E3Nic	34.9
Epi-nd	34.6
Epi/PNU-nd	33.7
EVP-dtg	34.3
EVP/PNU-dtg	34.3

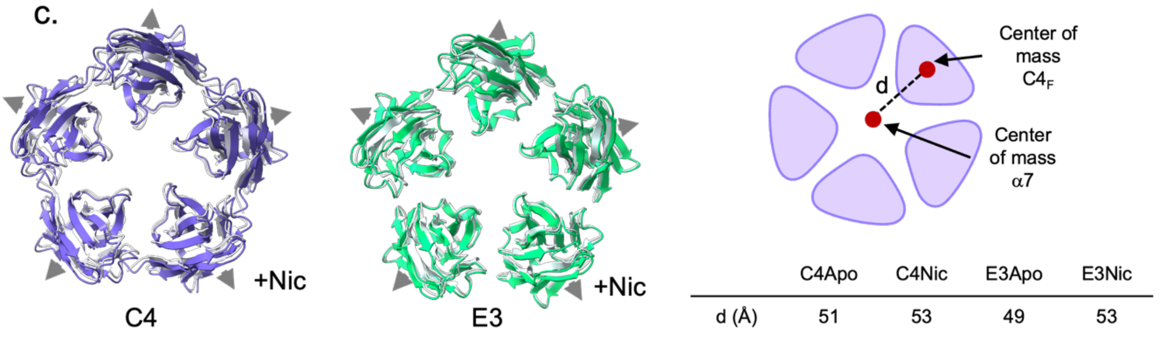

Supplementary figure 9: Tertiary and quaternary reorganization of nanobody-bound structures in the presence of nicotine

a. Tertiary variations of α7 between structures. Cα rmsd were calculated in ChimeraX using the C4-Nic structure as reference upon superimposition of monomers. ECD monomer of C4-Apo, E3-Nic and E3-Apo are shown in cartoons were Cα are colored according to the rmsd value, with the same range for all and shown in the color key.

b. ECD expansion between E3-Apo and E3-Nic. Distances are measured between the Cα of Ser69 from chain A and the center of mass of the Cα of the five Ser69 which figure the middle of the upper part of the ECD. The distance is shown on a top view of the E3-Apo (grey) and E3-Nic (pink) aligned and represented in ribbons. The table shows the values of this distance on several structures. Note that the expansion is absent between the two detergent structures and is similar for the two pairs of nanodisc structures.
c. Nanobodies expansion upon nicotine binding. With both nanobodies, we observed a small outward motion of the nanobodies molecules upon nicotine binding, as represented from the top with Apo structures in white and nicotine-bound structures in purple and green respectively. We found this motion to be around 2-3 Å by measuring the distance between the center of mass of one nanobody and the center of mass of α7 (summarized in the table).

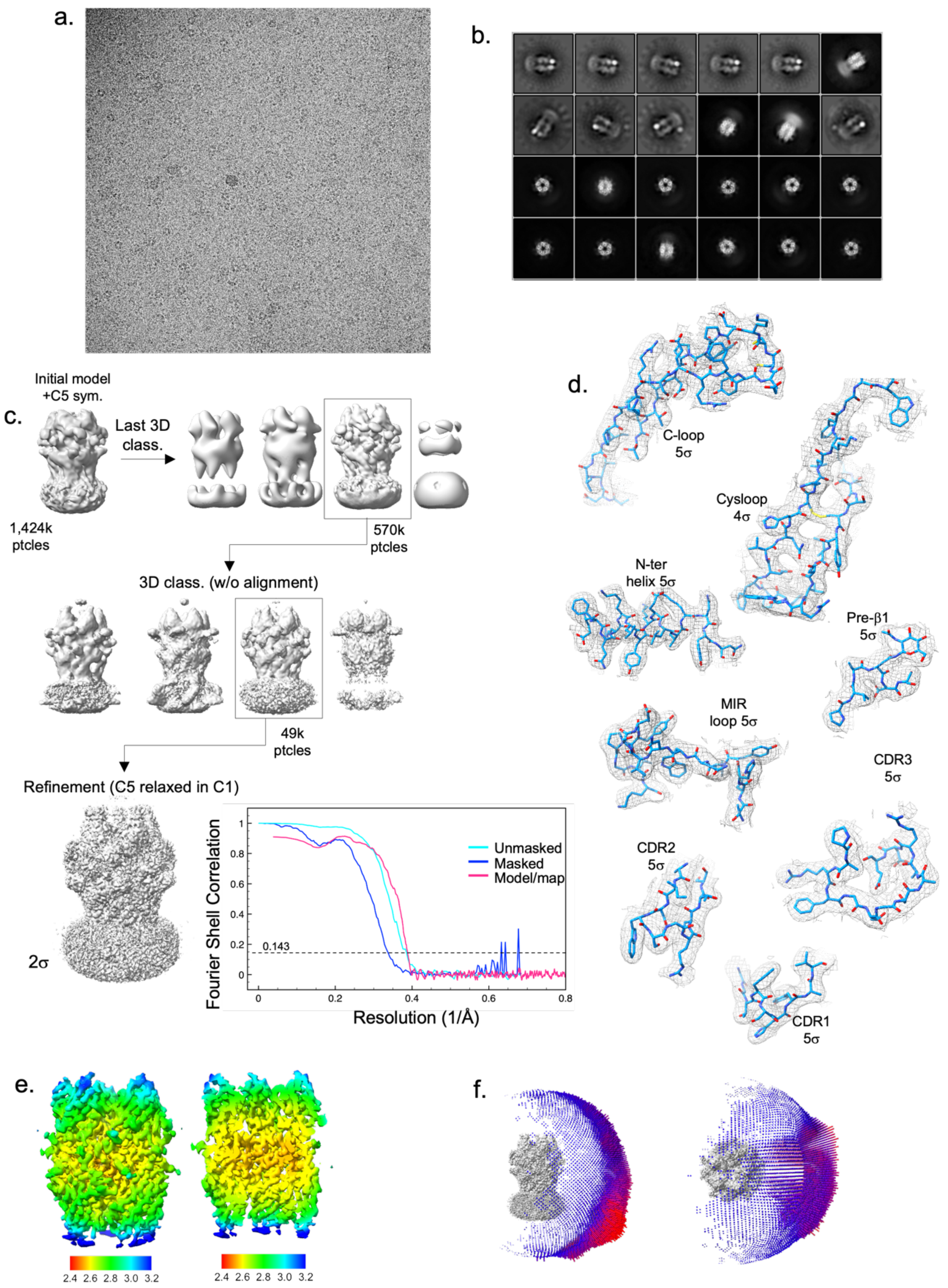

Supplementary figure 10: Electron microscopy and 3D reconstruction of the E3-Apo data set

a. Representative micrograph of the E3-Apo dataset.
b. Selected 2D class averages
c. Flowchart of the 3D volume processing. A C5-symmetry was applied on the initial model for 3D classification with particles alignment. The best class particles were submitted to another 3D classification round without alignment and high-resolution reaching particles further refined with a C5 symmetry relaxed in C1 (Relion 3D refine). Resulting volume is shown at 2σ contouring with the FSC curves on its right.

d. Representative densities and model building for the loop C, cys-loop, Nter helix, pre-β1, MIR of α7 and the three CDRs of E3 with the indicated contouring level.
e. Local resolution map calculated with Relion. The volume is shown at high contouring from the side and sliced in the vestibule.
f. Angular distribution of the particles seen on the side and top views of the unsharpened map.

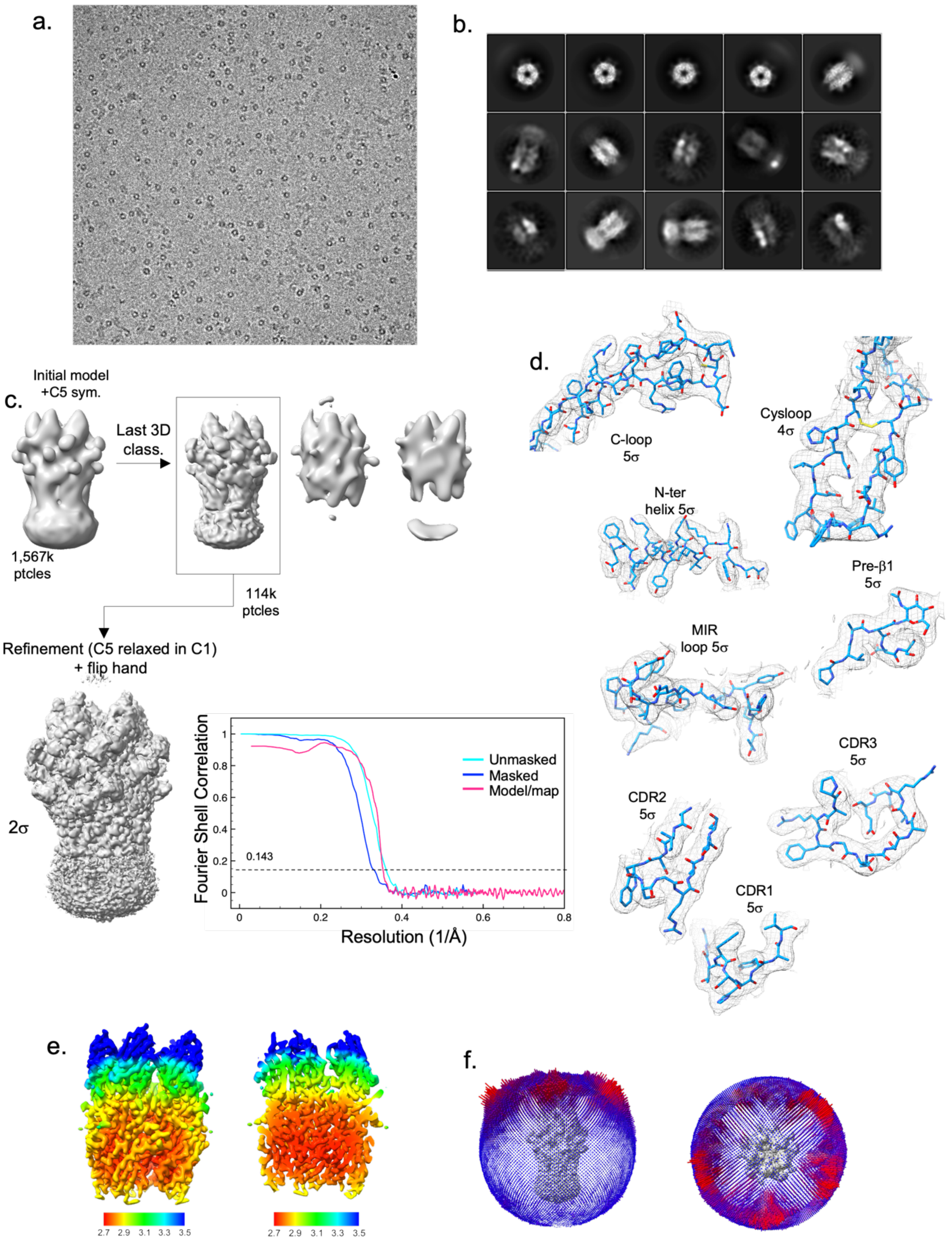

Supplementary figure 11: Electron microscopy and 3D reconstruction of the E3-Nic data set

a. Representative micrograph of the E3-Nic dataset.
b. Selected 2D class averages
c. Flowchart of the 3D volume processing. A C5-symmetry was applied on the initial model for 3D classification with particles alignment. The best class particles were further refined with a C5 symmetry relaxed in C1 (Relion 3D refine). Resulting volume is shown at 2σ contouring with the FSC curves on its right.

d. Representative densities and model building for the loop C, cys-loop, Nter helix, pre-β1, MIR of α7 and the three CDRs of E3 with the indicated contouring level.
e. Local resolution map calculated with Relion. The volume is shown at high contouring from the side and sliced in the vestibule.
f. Angular distribution of the particles seen on the side and top views of the unsharpened map.

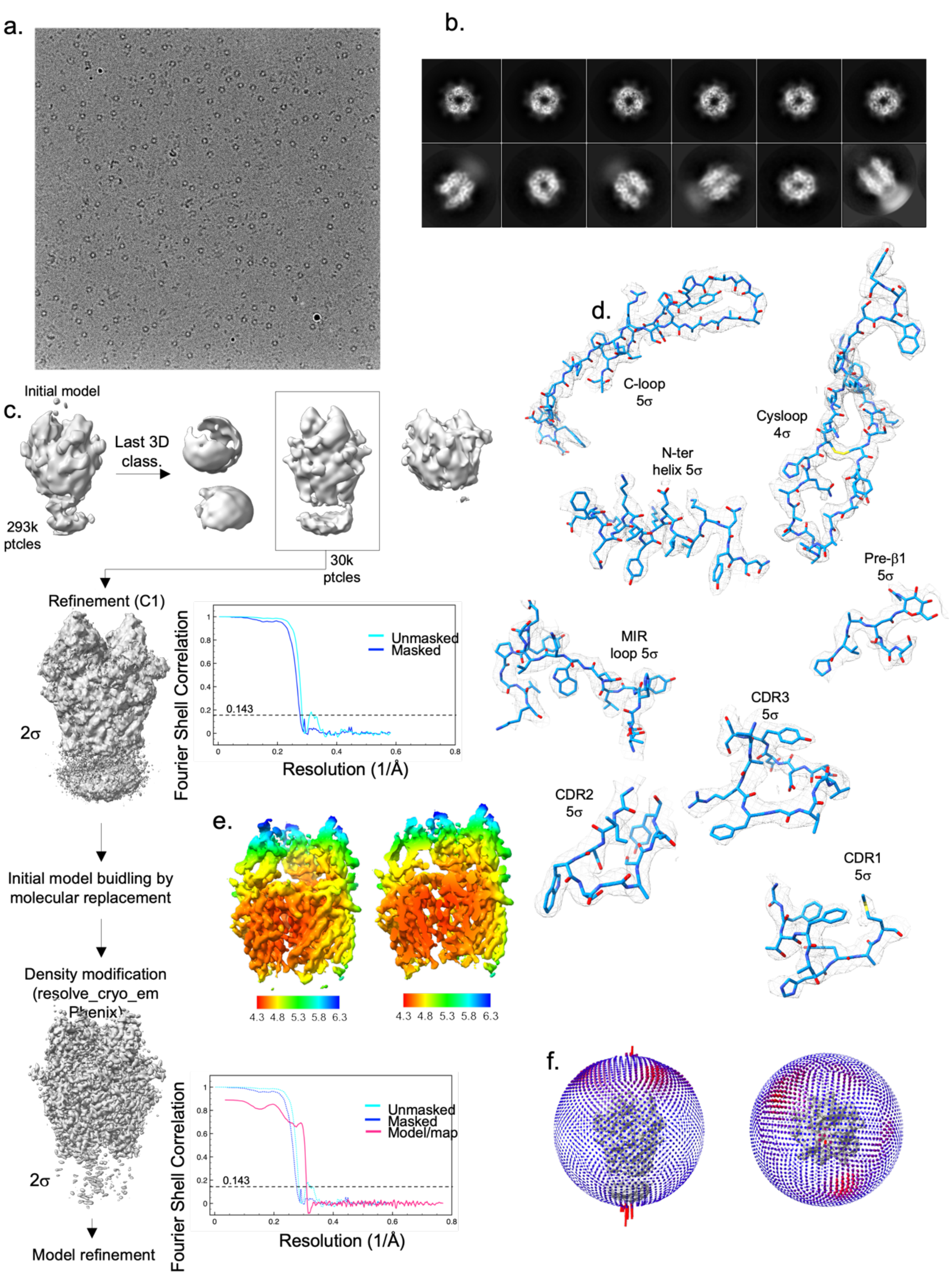

Supplementary figure 12: Electron microscopy and 3D reconstruction of the C4partial-Apo data set

a. Representative micrograph of the C4partial-Apo dataset
b. Selected 2D class averages
c. Flowchart of the 3D volume processing. No symmetry was applied on the initial model for 3D classification with particles alignment. The best class particles were further refined (Relion 3D refine). Resulting volume is shown at 2σ contouring with the FSC curves on its right. After a first model building, the density and the model were subjected to density modification in Phenix,

resulting in a volume allowing the refinement of a protein model at 3.4 resolution shown at 2σ contouring with the FSC curves on its right

d. Representative densities and model building for the loop C, cys-loop, Nter helix, pre-β1, MIR of α7 and the three CDRs of C4 with the indicated contouring level.

e. Local resolution map calculated with Relion. The volume is shown at high contouring from the side and sliced in the vestibule.

f. Angular distribution of the particles seen on the side and top views of the unsharpened map.

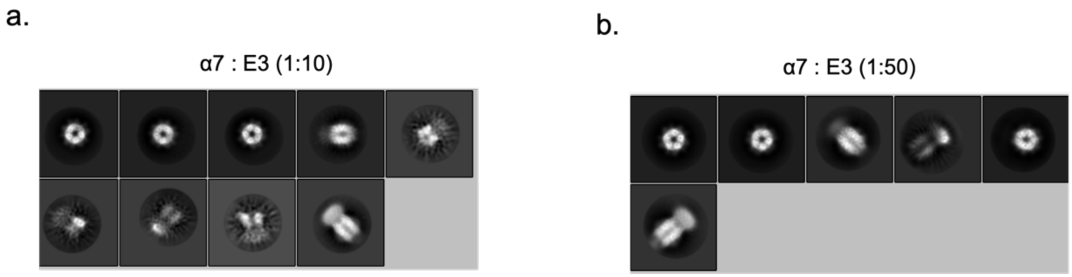

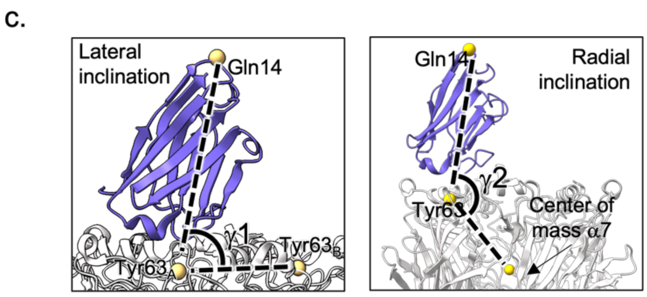

	C4-Nic F	C4-Apo F	C4partial F	C4partial G	C4partial I
Lateral γ1 (°)	85.3	85.1	82.8	80.5	78.2
Radial γ2 (°)	133.8	133.4	129.3	128.8	126.0
interface with α7 (Å²)	503.5	472.8	549.5	457.7	507.3

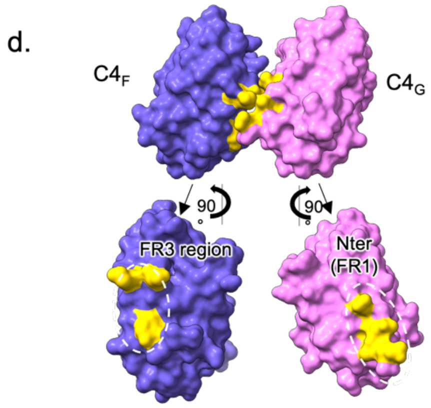

Supplementary figure 13: Analysis of the C4partial-Apo dataset

a. Preliminary work was done with lower concentration of E3 in the grids (10µM) and early 2D classes showed heterogeneity with most classes showing 4 molecules bound to α7.
b. Grids prepared the same day with 50µM E3 showed a vast majority of particles with 5 E3 molecules bound.
c. Orientation of the C4 molecules in the C4partial-Apo structure. The lateral inclination angle is defined between the Cα of Gln14 in C4, of Tyr63 in the α7 chain below and of Tyr63 of the complementary α7 subunit. The radial inclination angle is defined between the Cα of Gln14 on C4, of Tyr63 on the α7 subunit below and the center of mass of the α7 pentamer.
d. Surface representation of the two adjacent C4 (F in purple, G in pink) in the C4partial-Apo dataset and their exploded view below. Residues at the interface are colored in yellow.

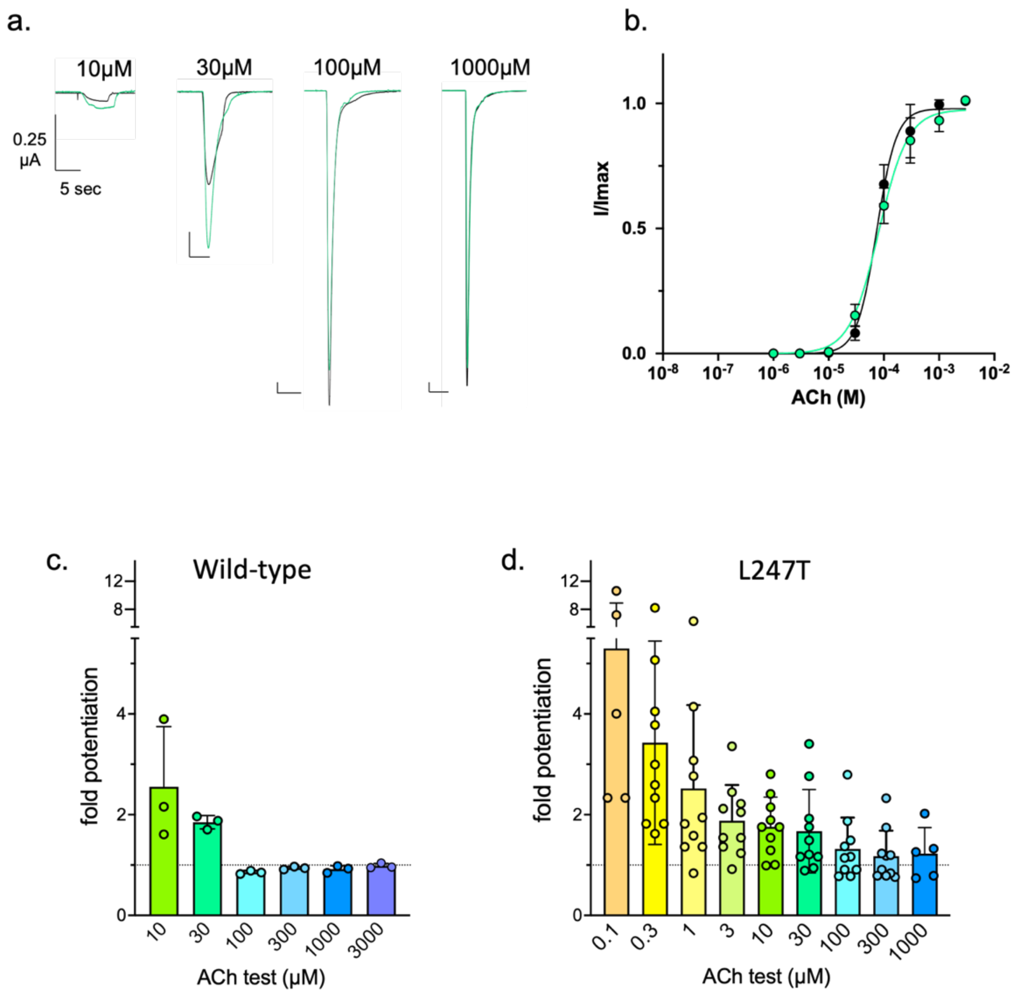

Supplementary Figure 14 : ACh concentration effect on E3 potentiation.

a. Representative traces of ACh dose-response curves of the α7 FLcryo with (green) or without (black) 15-seconds pre-application of 1µM E3 on a single cell.
b. Resulting dose-response curves after normalization on maximal currents of both conditions. Points are mean ± s.d. with n=3 cells.
c. Fold potentiation of α7 FLcryo currents after 15-seconds pre-application of 1µM E3 at various ACh concentrations. Points are mean ± sd with n=3 cells
d. Fold potentiation of α7 L247T mutants currents after 15-seconds pre-application of 1µM E3 at various ACh concentrations. Points are mean ±sd with n≥5 cells

.

	C4-Apo (EMDB-) (PDB)	C4-Nic (EMDB-(PDB	E3-Apo (EMDB) (PDB)	E3-Nic (EMDB) (PDB)	C4partial-Apo (EMDB) (PDB)
**Data collection and processing**					
Microscope	IC-Krios1 EMBL, Heidelberg	Titan-Krios IP, Paris	IC-Krios1 EMBL, Heidelberg	Titan-Krios IP, Paris	Glacios IP, Paris
Magnification	165 000	105 000	165 000	105 000	150 000
Voltage (kV)	300	300	300	300	200
Electron exposure (e–/Å^2)	40	40	40	40	50
Defocus range (μm)	-0.6 to -1.6	-0.6 to -2.2	-0.6 to -1.6	-0.6 to -2.2	-0.8 to -2.4
Pixel size (Å)	0.731	0.86	0.731	0.86	0.96
Symmetry imposed	C5	C5	C5	C5	C1
Movies	20151	5103	11090	5757	4506
Initial particle images (no.)	1534810	1064223	1424083	1566807	293673
Final particle images (no.)	104409	73690	49462	114385	29644
Map resolution (Å)	2.3	3.4	2.7	2.8	4.4
FSC threshold	0.143	0.143	0.143	0.143	0.143
Map resolution range (Å)	2.15-8.43	3.08-4.22	2.5-11.1	2.71-7.79	4.38-12
**Refinement**					
Initial model used (PDB code)					
Model resolution (Å)	2.46	3.51	2.53	2.7	3.4*
FSC threshold	0.143	0.143	0.143	0.143	0.143
Map sharpening *B* factor (Å^2)	-100	-100	-100	-100	-100
Model composition					
Non-hydrogen atoms	13465	13615	13010	13655	11145
Protein residues	1635	1635	1645	1660	1393
Ligands	NAG: 20	NCT:5 NAG: 20	NAG: 20	NCT: 5 NAG: 20	NAG: 20
B factors (Å^2)					
Protein	74.0	63.5	40.11	84.9	36.8
Ligand	86.2	60.1	48.64	100.0	51.4
R.m.s. deviations					
Bond lengths (Å)	0.004	0.003	0.004	0.003	0.004
Bond angles (°)	0.635	0.553	0.584	0.441	0.585
Validation					
MolProbity score	1.87	1.82	1.93	1.43	2.01
Clashscore	5.12	6.08	5.41	4.33	6.56
Poor rotamers (%)	2.46	0.14	2.39	0.7	0
Ramachandran plot					
Favored (%)	95.67	92.14	95.02	96.65	85.11
Allowed (%)	4.33	7.86	4.92	3.35	14.89
Disallowed (%)	0	0	0.06	0	0

*After density modification from Phenix[17]

Supplementary Table 1 Cryo-EM data collection, refinement and validation statistics

		R-like states			A/D-like states						
		C4Apo	Apo- (7EKI)	Bungaro (7KOO)	E3Apo	E3Nic	C4Nic	EVP (7EKP)	EVP+PNU (7EKT)	Epib+PNU (7KOX)	Epib (7KOQ)
R-like states	C4Apo		0.805	**0.595**	0.73	1.16	1.27	0.974	1.02	1.13	1.34
	Apo- (7EKI)			0.5	1.27	0.96	1	0.95	1.01	1.53	1.27
	Bungaro (7KOO)				1.18	1.11	1.22	1.04	1.09	1.42	1.29
A/D-like states	E3Apo					0.836	0.9	0.62	0.67	**0.454**	0.86
	E3Nic						0.29	0.62	0.54	1.03	**0.51**
	C4Nic							0.64	0.59	1.07	**0.56**
	EVP (7EKP)								0.23	0.83	0.64
	EVP+PNU (7EKT)									0.81	0.55
	Epib+PNU (7KOX)										0.85

Structures in detergent micelles

Structures in saposin/azolectin nanodiscs

Best match

Supplementary Table 2 : Cα rmsd values calculated on the pentamer of ECD for the indicated structures

			CDR1			CDR2	CDR3										
			Gly27	His32	Tyr33	Trp54	Arg101		Phe102		Gly103	Val104	Asp109			Ser111	Tyr112
			CO	NHε	OHη	NHε	CO	NHη	Bz	CO	N	Cγ	OH∂	N	CO	OHγ	OHη
Nter helix n+1	Glu1	N				+											
	Arg4	NHη								+	+						
	Lys5	Cγ										+					
		NHε															
	Lys8	NHε															
Nter helix	Glu9	OH∂			+											+	+
		CO															
	Lys12	N											+			+	
		NHε															
	Asn13	NH∂					+			+				+	+		
		OH∂											+	+			
Pre-β1	Asn23-NAG	COOH															
	Asn23	CO															
MIR	His62	NHε															
	Tyr63	CO						+									
		Bz							+								
	Gln65	CO						+									
		OHε		+													
	Glu70	CO															
		OHε	+														

Supplementary Table 3: Atomic contacts between C4 and α7.

Contacts were selected when the inter-atomic distance is bellow 4 Å , unless specified

Supplementary Table 4: Atomic contacts between E3 and α7.

			CDR1			CDR2			CDR3						
			Gly26	Gly27	Tyr32	Trp53	Arg56	Ser57	Arg100		Phe101		Asp109	Glu110	Asp112
			CO	N	OHη	Indol	NHη	OHγ	CO	NHη	Bz	CO	OH∂	CO	OH∂
Nter helix n+1	Glu1	N													
	Arg4	NHη								+		+			
	Lys5	Cγ													
		NHε						+							
	Lys8	NHε											+	+*	
Nter helix	Glu9	OH∂			+					+					
		CO							+						
	Lys12	N													
		NHε													+
	Asn13	NH∂								+				+	
		OH∂													
Pre-β1	Asn23-NAG	COOH					+								
	Asn23	CO					+								
MIR	His62	NHε				+									
	Tyr63	CO													
		Bz								+	+				
	Gln65	CO							+						
		OHε													
	Glu70	CO	+	+											
		OHε													

Contacts were selected when the inter-atomic distance is bellow 4 Å, unless specified
* 4.4 Å
